# Sexual Activity in Couples Dealing With Breast Cancer. A Cohort Study of Associations With Patient, Partner and Relationship-Related Factors

**DOI:** 10.3389/fpsyg.2022.828422

**Published:** 2022-04-07

**Authors:** Nina Rottmann, Pia Veldt Larsen, Christoffer Johansen, Mariët Hagedoorn, Susanne Oksbjerg Dalton, Dorte Gilså Hansen

**Affiliations:** ^1^Department of Psychology, University of Southern Denmark, Odense, Denmark; ^2^Research Unit of General Practice, Department of Public Health, University of Southern Denmark, Odense, Denmark; ^3^REHPA, The Danish Knowledge Center for Rehabilitation and Palliative Care, University Hospital Odense and Department of Clinical Research, University of Southern Denmark, Nyborg, Denmark; ^4^Mental Health Services in the Region of Southern Denmark, Vejle, Denmark; ^5^Oncology Department, Finsen Center, Rigshospitalet, University of Copenhagen, Copenhagen, Denmark; ^6^Survivorship and Inequality in Cancer, Danish Cancer Society Research Center, Copenhagen, Denmark; ^7^Health Sciences/Health Psychology, University Medical Center Groningen, University of Groningen, Groningen, Netherlands; ^8^Department of Clinical Oncology and Palliative Care, Zealand University Hospital, Næstved, Denmark; ^9^Department of Clinical Medicine, Faculty of Health and Medical Sciences, University of Copenhagen, Copenhagen, Denmark

**Keywords:** breast cancer, sexual activity, couples, patient, partner, relationship, cohort study

## Abstract

**Objective:**

Breast cancer may profoundly affect a couple’s sex life. The present study examines whether patient-, partner- and relationship-related characteristics are associated with sexual activity of couples following breast cancer diagnosis in the treatment phase and over time.

**Methods:**

Women with breast cancer and their male cohabiting partners participated in a longitudinal study in Denmark. Logistic regression was used to examine associations of patient-, partner- and relationship-related characteristics at baseline (≤4 months following surgery) with couples’ sexual activity at baseline, 5 and 12 months later. The longitudinal analyses were stratified for couples’ sexual activity status at baseline.

**Results:**

A total of 722, 533 and 471 couples were included in the analyses at baseline, 5- and 12-months follow-up, respectively. Older age, depressive symptoms and lower vitality of patients were associated with lower odds of couples’ sexual activity at baseline; chemotherapy treatment and older age of patients were associated with lower odds at 5-months follow-up in couples who were not sexually active at baseline. Higher ratings of emotional closeness, affectionate behavior and satisfaction with dyadic coping were associated with higher odds for sexual activity at baseline and over time in couples who were sexually active at baseline.

**Conclusion:**

Sexual counseling during cancer treatment and rehabilitation should include a couple perspective. Relationship-related variables may be a protective factor for remaining sexually active after breast cancer diagnosis. Interventions could focus on strengthening these factors. Health professionals also need to consider the patients’ breast cancer treatment, vitality, and emotional distress in counselling on sexuality.

## Introduction

According to the WHO sexuality is “a central aspect of being human throughout life” (World Health Organization, 2006, p. 5), and sexual activity has been found associated with greater enjoyment in life (Smith et al., 2019). For many cancer patients, including women with breast cancer, sexuality is a significant aspect of quality of life (Flynn et al., 2011; Bober and Varela, 2012). Yet, patients and their partners may experience changes to their sexual life after breast cancer diagnosis: The different changes that diagnosis and treatment of a potentially life-threatening illness may bring about, such as side or late effects of treatment, psychological distress and changed social roles can affect a couple’s sexual relationship (Fletcher et al., 2010; Ussher et al., 2012; Keesing et al., 2016). Couples may renegotiate their sexuality, and while some can experience a strengthened sexual relationship, others might less frequently engage in sexual activity or even cease to be sexually active. The literature on sexual activity following cancer is inconsistent, with some studies reporting decreases in sexual activity following cancer diagnosis (Ussher et al., 2012; Male et al., 2016), while others report sexual activity levels that are comparable to cancer-free controls (Jackson et al., 2016).

Sexuality within couples is a dyadic issue, and qualitative research among couples dealing with breast cancer has pointed out difficulties in resuming sexual activity for both patients and partners (Loaring et al., 2015; Keesing et al., 2016). Factors related to the patient, the partner and their experience of the couple relationship are likely to be relevant for couples’ sexual activity.

Previous studies have examined whether breast cancer treatment, emotional distress after breast cancer diagnosis (here defined as depressive symptoms) and other possible side or late effects of breast cancer treatment are associated with sexual activity among partnered breast cancer survivors. Treatment with chemotherapy has been found associated with sexual inactivity in some (Avis et al., 2018), but not all studies (Fobair et al., 2006), and several studies found no significant associations between sexual inactivity and other treatment modalities (Fobair et al., 2006; Marino et al., 2017; Avis et al., 2018). Findings suggest that depressive symptoms (Marino et al., 2017; Avis et al., 2018), self-image problems, such as not feeling attractive (Marino et al., 2017; Avis et al., 2018), and lack of vitality (Fobair et al., 2006) are associated with sexual inactivity. Cancer survivors have also reported pain as one of several reasons for decreases in sexual frequency (Ussher et al., 2015); among women with breast cancer, pain in the arm, breast and shoulder area is frequent (Gartner et al., 2009) and might impact sexual activity. Further, older age has been associated with sexual inactivity among women with breast cancer (Avis et al., 2018) and in the general population (Kleinstäuber, 2017), as have other medical or chronic conditions (Kleinstäuber, 2017).

It is likely that partners’ emotional distress after breast cancer diagnosis, their potential physical health problems and age also affect couples’ sexual activity (Kleinstäuber, 2017). In line with this, breast cancer survivors have reported physical problems of the partner as one of several reasons for sexual inactivity (Meyerowitz et al., 1999), and women who perceived their partner to fear sexual intercourse were found to be less likely to be sexually active (Brédart et al., 2011). However, these studies measured partner-related variables indirectly through the patients’ ratings and did not include the partners themselves. Thus, studies are needed that also include the partners’ perspective.

The role of relationship factors in couples’ sexual activity following breast cancer has been examined sparsely and primarily from the patients’ perspective. While emotional closeness to one’s partner was not associated with sexual activity in one study (Marino et al., 2017), breast cancer survivors’ feelings of emotional separation in the couple relationship were negatively associated with their sexual activity in another study (Brédart et al., 2011). Behavioral aspects in the relationship may also play a role for sexual activity. Findings from the broader literature on sexual health point towards the relevance of touching and physical closeness. However, only few studies have assessed such associations (Kleinstäuber, 2017); yet, studies point to the general importance of affectionate touch in relationships for both psychological, relational and physical wellbeing (Jakubiak and Feeney, 2017; Debrot et al., 2020). Finally, couples’ perceptions of how they deal with stress as a couple, more specifically, their satisfaction with their dyadic coping, may influence sexual activity (Bodenmann et al., 2010).

To be able to provide sexual counselling and support to couples during cancer treatment and rehabilitation it is central to understand what characterizes couples who are sexually active versus inactive after breast cancer diagnosis. However, studies that not only include the patients’ but also the partners’ perspective and that examine the role of relationship-related factors are sparse. Furthermore, previous research in this area has often been cross-sectional (Meyerowitz et al., 1999; Fobair et al., 2006; Brédart et al., 2011; Marino et al., 2017) and only few studies have used a longitudinal design (Avis et al., 2018). As challenges can occur in different phases throughout the cancer trajectory, a longitudinal perspective is important though. We need to know whether we early on in the cancer trajectory can identify factors that predict couples’ sexual activity at a later time point, when they transition through the often challenging re-entry phase, in which they may have to create a new normal and deal with the changes the breast cancer has brought about, to the early survivorship phase (Stanton et al., 2005).

The present study aims at contributing to filling these research gaps. Using an epidemiological approach and including information from both patients and partners within couples in a longitudinal design, this study will examine factors related to couples’ sexual activity, assessed as a couple-based outcome, while adjusting for a set of possible confounders (age, chemotherapy treatment and type of breast cancer surgery). We wish to identify potential risk or protective factors with respect to couples’ sexual activity that clinicians should pay attention to when they meet a couple, a patient or partner in a clinical setting. To our knowledge this is the first study on factors associated with couples’ sexual activity after breast cancer that has a longitudinal design and systematically includes the partner.

The overall aim of the present longitudinal study is to examine whether patient-, partner- and relationship-related characteristics are associated with sexual activity of couples following breast cancer diagnosis in the treatment phase and over time. Firstly, we examine whether individual characteristics of patients and partners and their experience of the couple-relationship in the treatment phase (Time1, T1) are associated with couples’ sexual activity at T1. Secondly, we examine whether individual characteristics of patients and partners and their experience of the couple-relationship in the treatment phase (T1) are associated with couples’ sexual activity at the re-entry phase (Time 2, T2) and the early survivorship phase (Time 3, T3), stratified on sexual activity at T1.

## Materials and Methods

The present study is based on data from the Danish Couples and Breast Cancer Cohort (DCBCC; Terp et al., 2015), a nationwide, population-based cohort of couples dealing with breast cancer in Denmark. It includes self-report data from both patients and partners and data from Danish nationwide registries.

### Procedure and Participants

Between July 2011 and August 2012, all women newly diagnosed with breast cancer who were cohabiting with a male partner in Denmark were eligible to participate. Specifically, couples were eligible if the patient was female, aged ≥18 years, residing in Denmark, had had surgery for primary invasive breast cancer no more than 4 months before study invitation, and was cohabiting with a male partner aged ≥18 years.

Couples received questionnaires at baseline (≤4 months following surgery; T1), and 5 months (T2) and 12 months (T3) later, which assessed their individual wellbeing and relationship aspects. Demographic and health-related data were obtained from nationwide administrative, health- and disease-specific registries. The study procedure has been described in detail elsewhere (Terp et al., 2015).

In the present study, we used self-report data on couples’ sexual activity at baseline, T2 and T3. All other included data were assessed at baseline. We excluded couples with missing information on sexual activity at baseline.

The study was reported to the Danish Data Protection Agency *via* the University of Southern Denmark (file number SDU 10.143). The project was also notified to The Regional Scientific Ethical Committee for Southern Denmark, which assessed that the project fell outside the scope of projects to be approved by an Ethical Committee in Denmark (ID: S-20110103).

### Measurements

Unless otherwise specified all measures were obtained for both patients and partners.

#### Sexual Activity

Sexual activity was assessed using one item from the Patient-Reported Outcomes Measurement Information System (PROMIS)^®^ Sexual Function and Satisfaction measure (SexFS) version 1.0 (Flynn et al., 2013): *‘In the past 30 days, when you have had sexual activity, how satisfying has it been?’*. Response options ranged from 1 = *not at all* to 5 = *very* and included the option 0 = *have not been sexually active in the past 30 days*. A respondent was categorized as sexually active if she/he chose one of the response options *not at all* to *very* (satisfying) on the sexual satisfaction item. A respondent was categorized as not sexually active if she/he reported that she/he had not been sexually active in the past 30 days. Patients’ and partners’ scores were then combined in a couple score: Couples were considered as not sexually active, if one partner or both had been categorized as not sexually active in the past 30 days. Couples were scored as active if both partners had been categorized as sexually active in the past 30 days.

#### Depressive Symptoms

Depressive symptoms were measured using the Danish version of the Center for Epidemiologic Studies-Depression Scale (CES-D), a 20-item validated scale assessing depressive symptoms in the last week (Radloff, 1977; Hann et al., 1999). Higher scores indicate more symptoms (score range: 0–60). In the present study, Cronbach’s alpha was 0.9 for patients and partners in our sample.

##### Pain

Patients’ pain in the arm, breast or shoulder area was assessed with a single item inspired by the item format of the Breast Cancer Prevention Trial Eight Symptom Scale (BESS; Cella et al., 2008). Patients were asked to report to which degree they had been bothered by pain in the arm, breast, or shoulder area in the past 4 weeks on a five-point scale with response options ranging from *0 = not at all* to 4 *= extremely*.

##### Vitality

Patients’ vitality was measured by the four-item vitality subscale of the SF-36® Health Survey (version 1) with 6-point rating scales (Bjørner et al., 1997, 1998; Ware and Gandek, 1998). The scale score ranges from 0 to 100 with higher scores indicating higher vitality. The scale has previously been used as an indicator of fatigue (Brown et al., 2011). Cronbach’s alpha was 0.87.

##### Body Image

Patients indicated body image symptoms on the 10-item Body Image Scale (Hopwood et al., 2001). Items have four response options ranging from *0 = not at all* to *3 = very much*. The total score ranges from 0–30 with higher scores indicating greater body image disturbance. Cronbach’s alpha was 0.87.

#### Emotional Closeness

Emotional closeness was measured by one item on how close participants felt to their partner during the past 30 days [inspired by Manne et al. (2004)]. Response options ranged from *1 = not at all* to *5 = very*, thus higher scores represent a higher degree of emotional closeness.

#### Affectionate Behavior

Affectionate behavior was measured with two items from the PROMIS^®^ SexFS version 1.0 item pool on sexual activities: one item on the frequency of holding and hugging romantically, and one on the frequency of kissing romantically, with another person in the past 30 days (Flynn et al., 2013). We replaced ‘another person’ by ‘your partner’. The response options for both items included *1 = have not done in the past 30 days; 2 = once a week or less; 3 = once every few days; 4 = once a day;* and *5 = more than once a day*. As the two items were highly correlated within patients (*r* = 0.76) and partners (*r* = 0.79), mean scores were computed as a single score for affectionate behavior (range 1–5).

#### Satisfaction With Dyadic Coping

Satisfaction with dyadic coping was assessed with the Evaluation of dyadic coping-subscale of the Dyadic Coping Inventory (DCI; Bodenmann, 2008). Respondents rate their satisfaction with the couple’s dyadic coping in times of stress on two items with five response options each (*1 = very rarely; 5 = very often*). A score is computed by summing the two items (scale range: 2–10). The two items were highly correlated: *r* = 0.91 within both patients and partners.

#### Demographic and Health-Related Information

We obtained information on age at time of study invitation through the Danish Civil Registration System (Pedersen, 2011) and information on breast cancer treatment through the database of the Danish Breast Cancer Cooperative Group (Moller et al., 2008). Relationship length was self-reported by the patient at T1. Based on data from the Danish National Patient Register, covering all hospitalizations since 1977 and outpatient visits since 1995 (Lynge et al., 2011), we calculated the Charlson Comorbidity Index (CCI; Charlson et al., 1987) as a measure of patient comorbidity and partner morbidity. The CCI includes 19 different conditions, such as myocardial infarction, chronic pulmonary disease, and diabetes. Patients’ breast cancer diagnosis was not included in the CCI.

### Statistical Analyses

#### Descriptive Statistics

We calculated percentages or mean values with standard deviations (SD) for sample characteristics. The Pearson correlations within couples were calculated for the relationship-related variables satisfaction with dyadic coping, emotional closeness and affectionate behavior that were rated by both patients and partners. The agreement between patient and partner on ratings of sexual activity was evaluated using Cohen’s kappa and % of agreement.

#### Inferential Statistics

To examine the association between couples’ demographic, health-, quality of life- and relationship-related characteristics at baseline and their sexual activity at baseline, T2 and T3, respectively, we used logistic regression. Odds ratios (ORs) of continuous covariates are presented per scale unit as well as per sample standard deviation (SD) of the given covariate. All regression analyses were adjusted for age, type of surgery and chemotherapy. The longitudinal analyses, which included couples’ sexual activity at T2 and T3 as outcome, were stratified on couples’ sexual activity at baseline in separate models. Further, for all relationship-related variables with ratings by both partners, sensitivity analyses were conducted with additional adjustment for the respective partner’s score. For all models, assumptions on linearity of continuous covariates were assessed using deviance residual plots. Due to indications that the linearity assumption on affectionate behavior was not satisfied at T1, the variable was dichotomized in all analyses. Based on the content of the response options a score of <3 was defined as infrequent and a score of ≥3 as frequent affectionate behavior.

Because of the large number of analyses only results at a significance level of *p* < 0.01 are described and discussed.

## Results

### Study Sample

A total of 2,254 couples were eligible for the DCBCC study, and 792 (35%) participated. The present study was based on the 722 couples (response rate: 32%), in whom information on sexual activity status at baseline (T1) was available for both patients and partners. Of these 722 couples, 533 (response rate: 24%) and 471 (response rate: 21%) also gave information on the study outcome at T2 and T3, respectively. Thus, of the 722 responders at T1, 74% had data available at T2, and 65% had data at T3.

At T1, non-responding couples (*n* = 70, 9%) were about a decade older (mean age patients: 67 vs. 57 years; partners: 70 vs. 59 years), fewer patients in non-responding couples had received chemotherapy (29% vs. 53%), and more partners had one or more comorbidities (53% vs. 35%). At T2 and T3, the characteristics of non-responders and responders were similar (data not shown).

The 722 couples in the present analysis were on average in their late fifties (see Table 1) and had been together for an average of 28.6 years (SD = 14.6). One quarter of the women (26%) had received a mastectomy, and half of them (53%) were allocated to chemotherapy treatment.

**Table 1:** Baseline patient and partner characteristics of 722 couples, stratified on couple sexual activity.

	Total	Not sexually active at baseline	Sexually active at baseline
Total, *n* (%)	722	293 (41%)	429 (59%)
**PATIENT**			
**Demographic and health characteristics**			
Age in years, mean (SD)	57.1 (10.4)	59.5 (10.3)	55.5 (10.3)
Charlson Comorbidity Index, *n* (%)			
No comorbidity (0)	520 (72)	201 (69)	319 (74)
Comorbidity (≥1)	202 (28)	92 (31)	110 (26)
**Breast cancer treatment**			
Type of surgery, *n* (%)			
Mastectomy	186 (26)	73 (25)	113 (26)
Lumpectomy	536 (74)	220 (75)	316 (74)
Allocated to chemotherapy, *n* (%)			
No	339 (47)	153 (52)	186 (43)
Yes	383 (53)	140 (48)	243 (57)
Allocated to endocrine treatment, *n* (%)			
No	200 (28)	85 (29)	115 (27)
Yes	522 (72)	208 (71)	314 (73)
**Quality of life-related factors**			
Depressive symptoms, mean (SD)^a^	12.0 (9.0)	13.3 (9.3)	11.0 (8.7)
Pain in arm, breast or shoulder area, mean (SD)^b^	0.9 (0.9)	0.9 (1.0)	0.9 (0.9)
Vitality, mean (SD)^c^	52.9 (22.3)	48.7 (21.8)	55.8 (22.3)
Body image, mean (SD)^d^	8.2 (6.0)	8.0 (6.1)	8.4 (6.0)
**Relationship-related factors**			
Satisfaction with dyadic coping, mean (SD)^e^	7.9 (1.9)	7.5 (2.2)	8.2 (1.7)
Emotional closeness, mean (SD)^f^	4.4 (0.8)	4.2 (0.9)	4.6 (0.7)
Affectionate behavior, *n* (%)^g^			
Infrequent (<3)	173 (24)	105 (36)	68 (16)
Frequent (≥3)	545 (76)	185 (64)	360 (84)
**PARTNER**			
**Demographic and health characteristics**			
Age, mean (SD)	59.1 (10.9)	61.7 (10.5)	57.3 (10.8)
Charlson Comorbidity Index, *n* (%)			
No comorbidity (0)	468 (65)	175 (60)	293 (68)
Comorbidity (≥1)	254 (35)	118 (40)	136 (32)
**Quality of life-related factors**			
Depressive symptoms, mean (SD)^h^	8.8 (8.3)	9.6 (9.1)	8.3 (7.6)
**Relationship-related factors**			
Satisfaction with dyadic coping, mean (SD)^i^	7.9 (1.9)	7.6 (2.1)	8.1 (1.7)
Emotional closeness, mean (SD)^j^	4.5 (0.8)	4.3 (1.0)	4.6 (0.6)
Affectionate behavior, *n* (%)^k^			
Infrequent (<3)	173 (24)	110 (38)	63 (15)
Frequent (≥3)	546 (76)	182 (63)	364 (85)

*SD, Standard deviation*.

a *3 missing values (0.4%)*.

b *4 missing values (0.6%)*.

c *1 missing value (0.1%)*.

d *4 missing values (0.6%)*.

e *19 missing values (2.6%)*.

f *3 missing values (0.4%)*.

g *4 missing values (0.6%)*.

h *2 missing values (0.3%)*.

i *25 missing values (3.5%)*.

j *1 missing value (0.1%)*.

k *3 missing values (0.4%)*.

A total of 59, 61 and 62% were sexually active at T1, T2 and T3, respectively. For most couples, sexual activity status did not change from T1 to T2 and from T1 to T3. From T1 to T2, 11% of couples changed from being not sexually active to being active, while 7% changed from being sexually active to not active. A similar pattern was observed from T1 to T3 (see Table 2).

**Table 2:** Couple sexual activity over time from baseline to T2 and T3, respectively.

From T1 to T2			From T1 to T3		
*N* = 533 couples			*N* = 471 couples		
Sexual activity status			Sexual activity status		
T1	T2	*n* (%)^a^	T1	T3	*n* (%)
Not active	Not active	154 (29)	Not active	Not active	124 (26)
Not active	Active	56 (11)	Not active	Active	57 (12)
Active	Not active	36 (7)	Active	Not active	24 (5)
Active	Active	287 (54)	Active	Active	266 (57)

*T1, Time 1, ≤4 months following surgery for breast cancer; T2, Time 2, 5 months; T3, Time 3, 12 months later*.

a *Percentages do not add up to 100 due to rounding*.

The agreement between patient and partner on sexual activity was high at all three timepoints (all % of agreement >83%), although kappa values were only moderate (all kappa ≥0.59). Patients’ and partners’ ratings of the relationship-related variables were moderately to strongly correlated: satisfaction with dyadic coping, *r* = 0.37; emotional closeness, *r* = 0.45; affectionate behavior, *r* = 0.64.

### Associations With Sexual Activity at Baseline

At baseline, older age and depressive symptoms of patients were significantly associated with lower adjusted odds for couples being sexually active (Table 3). The odds increased significantly with higher ratings of patient vitality and with higher ratings of all three relationship variables: satisfaction with dyadic coping, emotional closeness and affectionate behavior of both patients and partners.

**Table 3:** Associations between patient and partner characteristics and sexual activity at baseline.

	OR_crude_	OR_adj_ (95%-CI)^a^	OR_SDadj_ (95%-CI)^a^
**PATIENT**			
**Demographic and health characteristics**			
Age (per year)	0.96^***^	0.96 (0.94, 0.98)^***^	0.65 (0.53, 0.81)^***^
Charlson Comorbidity Index (ref. no comorbidity (0)): comorbidity (≥1)	0.75	0.85 (0.61, 1.19)	0.85 (0.61, 1.19)
**Breast cancer treatment**			
Type of surgery (ref. mastectomy): lumpectomy	0.93	1.01 (0.71, 1.43)	1.01 (0.71, 1.43)
Allocated to chemotherapy (ref. no): yes	1.43^*^	0.86 (0.59, 1.25)	0.86 (0.59, 1.25)
Allocated to endocrine treatment (ref. no): yes	1.12	1.13 (0.80, 1.58)	1.13 (0.80, 1.58)
**Quality of life-related factors**			
Depressive symptoms^b^	0.97^**^	0.96 (0.95, 0.98)^***^	0.69 (0.63, 0.83)^***^
Pain in arm, breast or shoulder area^b^	0.94	0.91 (0.77, 1.07)	0.92 (0.79, 1.06)
Vitality^b^	1.01^***^	1.02 (1.01, 1.03)^***^	1.55 (1.25, 1.93)^***^
Body image^b^	1.01	0.99 (0.96, 1.01)	0.94 (0.78, 1.06)
**Relationship-related factors**			
Satisfaction with dyadic coping^b^	1.19^***^	1.22 (1.12, 1.32)^***^	1.46 (1.24, 1.69)^***^
Emotional closeness^b^	1.75^***^	1.92 (1.56, 2.35)^***^	1.69 (1.43, 1.98)^***^
Affectionate behavior [ref. infrequent (<3)]: frequent (≥3)	3.00^***^	3.16 (2.19, 4.55)^***^	3.16 (2.19, 4.55)^***^
**PARTNER**			
**Demographic and health characteristics**			
Age (per year)	0.96^***^	0.97 (0.94, 1.01)	0.72 (0.51, 1.11)
Charlson Comorbidity Index [ref. no comorbidity (0)]: comorbidity (≥1)	0.69^*^	0.82 (0.60, 1.13)	0.82 (0.60, 1.13)
**Quality of life-related factors**			
Depressive symptoms^b^	0.98^*^	0.98 (0.96, 0.99)^*^	0.85 (0.71, 0.92)^*^
**Relationship-related factors**			
Satisfaction with dyadic coping^b^	1.13^**^	1.17 (1.07, 1.27)^***^	1.35 (1.14, 1.57)^***^
Emotional closeness^b^	1.69^***^	1.93 (1.56, 2.38)^***^	1.69 (1.43, 2.00)^***^
Affectionate behavior [ref. infrequent (<3)]: frequent (≥3)	3.49^***^	3.76 (2.60, 5.43)^***^	3.76 (2.60, 5.43)^***^

*OR, Odds Ratio; OR_SD_, Odds Ratio measured in units of sample SD for continuous covariates; ref., reference category*.

a *Adjusted for patient age, type of surgery and chemotherapy*.

b *Per scale unit (resp. SD) increase*.

* *p* *< 0.05;*

** *p* *< 0.01*

*** *p < 0.001*.

### Associations With Sexual Activity at Follow-Up

#### Couples Who Were Not Sexually Active at Baseline

Patients’ older age and chemotherapy treatment were significantly associated with lower odds for couples being sexually active at T2 (Table 4). No significant associations were found between couples’ baseline characteristics and their sexual activity at T3 (Table 5).

**Table 4:** Associations between patient and partner characteristics and sexual activity at time T2, stratified on baseline sexual activity.

	Associations with sexual activity at T2 (*n* = 533 couples)					
	Couples not sexually active at T1			Couples sexually active at T1		
	*n* = 210			*n* = 323		
	OR_crude_	OR_adj_ (95%-CI)^a^	OR_SDadj_ (95%-CI)^a^	OR_crude_	OR_adj_ (95%-CI)^a^	OR_SDadj_ (95%-CI)^a^
**PATIENT**						
**Demographic and health characteristics**						
Age (per year)	0.97	0.93 (0.89, 0.97)^**^	0.47 (0.30, 0.73)^**^	1.02	1.02 (0.97, 1.06)	1.23 (0.73, 1.82)
Charlson Comorbidity Index [ref. no comorbidity (0)]: comorbidity (≥1)	0.43^*^	0.43 (0.20, 0.93)^*^	0.43 (0.20, 0.93)^*^	1.00	0.99 (0.44, 2.24)	0.99 (0.44, 2.24)
**Breast cancer treatment**						
Type of surgery (ref. mastectomy): lumpectomy	1.01	1.04 (0.49, 2.24)	1.04 (0.49, 2.24)	1.34	1.19 (0.54, 2.60)	1.19 (0.54, 2.60)
Allocated to chemotherapy (ref. no): yes	0.58	0.24 (0.10, 0.56)^**^	0.24 (0.10, 0.56)^**^	0.65	0.82 (0.33, 2.03)	0.82 (0.33, 2.03)
Allocated to endocrine treatment (ref. no): yes	1.50	1.84 (0.86, 3.93)	1.84 (0.86, 3.93)	1.57	1.63 (0.78, 3.43)	1.63 (0.78, 3.43)
**Quality of life-related factors**						
Depressive symptoms^b^	1.00	1.00 (0.96, 1.04)	1.00 (0.68, 1.44)	1.00	1.01 (0.96, 1.05)	1.09 (0.70, 1.53)
Pain in arm, breast or shoulder area^b^	0.96	0.86 (0.61, 1.22)	0.86 (0.61, 1.22)	0.90	0.92 (0.63, 1.34)	0.93 (0.66, 1.30)
Vitality^b^	1.00	1.01 (0.99, 1.02)	1.24 (0.80, 1.54)	1.00	0.99 (0.98, 1.01)	0.80 (0.64, 1.25)
Body image^b^	0.98	0.94 (0.88, 1.01)	0.69 (0.46, 1.06)	0.98	1.00 (0.94, 1.06)	1.00 (0.69, 1.42)
**Relationship-related factors**						
Satisfaction with dyadic coping^b^	1.14	1.20 (1.01, 1.42)^*^	1.49 (1.02, 2.16)^*^	1.28^*^	1.28 (1.04, 1.58)^*^	1.52 (1.07, 2.18)^*^
Emotional closeness^b^	1.07	1.20 (0.82, 1.74)	1.18 (0.84, 1.65)	2.01^**^	2.05 (1.31, 3.21)^**^	1.65 (1.21, 2.26)^**^
Affectionate behavior [ref. infrequent (<3)]: frequent (≥3)	1.62	1.85 (0.90, 3.80)	1.85 (0.90, 3.80)	6.48^***^	6.93 (3.19, 15.09)^***^	6.93 (3.19, 15.09)^***^
**PARTNER**						
**Demographic and health characteristics**						
Age (per year)	0.98	1.04 (0.96, 1.11)	1.51 (0.65, 2.99)	1.03	1.04 (0.96, 1.14)	1.53 (0.64, 4.12)
Charlson Comorbidity Index [ref. no comorbidity (0)]: comorbidity (≥1)	0.40^**^	0.46 (0.22, 0.92)^*^	0.46 (0.22, 0.92)*	3.12^*^	3.02 (1.11, 8.20)^*^	3.02 (1.11, 8.20)^*^
**Quality of life-related factors**						
Depressive symptoms^b^	0.98	0.98 (0.94, 1.02)	0.98 (0.94, 1.02)	0.97	0.98 (0.93, 1.02)	0.86 (0.58, 1.16)
**Relationship-related factors**						
Satisfaction with dyadic coping^b^	1.11	1.14 (0.97, 1.34)	1.32 (0.94, 1.85)	1.39^***^	1.38 (1.16, 1.65)^***^	1.73 (1.29, 2.34)^***^
Emotional closeness^b^	1.45	1.59 (1.04, 2.43)^*^	1.59 (1.04, 2.43)^*^	1.93^**^	1.91 (1.20, 3.06)^**^	1.47 (1.12, 1.96)^**^
Affectionate behavior [ref. infrequent (<3)]: frequent (≥3)	2.05^*^	2.38 (1.17, 4.84)^*^	2.38 (1.17, 4.84)^*^	3.69^**^	3.92 (1.77, 8.69)^**^	3.92 (1.77, 8.69)^**^

*Odds ratios are shown with 95% confidence intervals (CI). OR, Odds Ratio; OR_SD_, Odds Ratio measured in units of sample SD for continuous covariates; ref., reference category;*

a *Adjusted for patient age, type of surgery and chemotherapy*.

b *Per scale unit (resp. SD) increase*.

* *p* *< 0.05;*

** *p* *< 0.01;*

*** *p < 0.001*.

**Table 5:** Associations between patient and partner characteristics and sexual activity at time T3, stratified on baseline sexual activity.

	Associations with sexual activity at T3 (*n* = 471 couples)					
	Couples not sexually active at time T1			Couples sexually active at time T1		
	*n* = 181			*n* = 290		
	OR_crude_	OR_adj_ (95%-CI)^a^	OR_SDadj_ (95%-CI)^a^	OR_crude_	OR_adj_ (95%-CI)^a^	OR_SDadj_ (95%-CI)^a^
**PATIENT**						
**Demographic and health characteristics**						
Age (per year)	0.99	0.98 (0.94, 1.03)	0.81 (0.53, 1.36)	1.00	1.00 (0.95, 1.05)	1.00 (0.59, 1.65)
Charlson Comorbidity Index [ref. no comorbidity (0)]: comorbidity (≥1)	0.61	0.62 (0.30, 1.25)	0.62 (0.30, 1.25)	0.75	0.74 (0.29, 1.92)	0.74 (0.29, 1.92)
**Breast cancer treatment**						
Type of surgery (ref. mastectomy): lumpectomy	1.36	1.37 (0.65, 2.92)	1.37 (0.65, 2.92)	0.80	0.77 (0.27, 2.19)	0.77 (0.27, 2.19)
Allocated to chemotherapy (ref. no): yes	1.08	0.91 (0.42,2.00)	0.91 (0.42,2.00)	0.88	0.85 (0.30, 2.45)	0.85 (0.30, 2.45)
Allocated to endocrine treatment (ref. no): yes	0.61	0.61 (0.30, 1.23)	0.61 (0.30, 1.23)	1.82	1.85 (0.78, 4.37)	1.85 (0.78, 4.37)
**Quality of life-related factors**						
Depressive symptoms^b^	0.99	0.99 (0.95, 1.02)	0.91 (0.62, 1.20)	0.98	0.98 (0.93, 1.02)	0.84 (0.53,1.19)
Pain in arm, breast or shoulder area^b^	0.82	0.83 (0.58, 1.18)	0.83 (0.58, 1.18)	0.95	0.94 (0.59, 1.50)	0.95 (0.62, 1.44)
Vitality^b^	1.01	1.01 (0.99, 1.02)	1.24 (0.80, 1.54)	1.00	1.00 (0.98, 1.02)	1.00 (0.64, 1.56)
Body image^b^	1.00	1.00 (0.94, 1.07)	1.00 (0.69, 1.51)	0.98	0.98 (0.91, 1.06)	0.89 (0.57, 1.42)
**Relationship-related factors**						
Satisfaction with dyadic coping^b^	1.03	1.04 (0.89, 1.21)	1.09 (0.77, 1.52)	1.32^*^	1.38 (1.06, 1.80)^*^	1.73 (1.10, 2.72)^*^
Emotional closeness^b^	1.12	1.14 (0.80, 1.63)	1.13 (0.82, 1.55)	1.53	1.59 (0.96, 2.65)	1.38 (0.97, 1.98)
Affectionate behavior [ref. infrequent (<3)]: frequent (≥3)	1.47	1.42 (0.70, 2.87)	1.42 (0.70, 2.87)	4.69^**^	4.90 (2.00. 12.02)^**^	4.90 (2.00. 12.02)^**^
**PARTNER**						
**Demographic and health characteristics**						
Age (per year)	0.99	1.01 (0.95, 1.09)	1.11 (0.58, 2.47)	1.00	0.99 (0.89, 1.09)	0.90 (0.28, 2.54)
Charlson Comorbidity Index [ref. no comorbidity (0)]: comorbidity (≥1)	0.69	0.71 (0.36, 1.38)	0.71 (0.36, 1.38)	1.48	1.51 (0.56, 4.05)	1.51 (0.56, 4.05)
**Quality of life-related factors**						
Depressive symptoms^b^	0.98	0.98 (0.95, 1.02)	0.83 (0.63, 1.20)	1.02	1.02 (0.96, 1.08)	1.16 (0.73, 1.79)
**Relationship-related factors**						
Satisfaction with dyadic coping^b^	1.14	1.15 (0.98, 1.36)	1.34 (0.96, 1.91)	1.37^**^	1.36 (1.11, 1.67)^**^	1.69 (1.19, 2.39)^**^
Emotional closeness^b^	1.27	1.30 (0.88, 1.91)	1.30 (0.88, 1.91)	2.08^**^	2.11 (1.26, 3.52)^**^	1.57 (1.15, 2.13)^**^
Affectionate behavior [ref. infrequent (<3)]: frequent (≥3)	1.98^*^	2.04 (1.03, 4.06)^*^	2.04 (1.03, 4.06)^*^	4.37^**^	4.39 (1.74, 11.04)^**^	4.39 (1.74, 11.04)^**^

*Odds ratios are shown with 95% confidence intervals (CI). OR, Odds Ratio; OR_SD_, Odds Ratio measured in units of sample SD for continuous covariates; ref., reference category*

a *Adjusted for patient age, type of surgery and chemotherapy*.

b *Per scale unit (resp. SD) increase*.

* *p* *< 0.05;*

** *p < 0.01*.

#### Couples Who Were Sexually Active at Baseline

Patients’ perception of emotional closeness and affectionate behavior, and partners’ perception of emotional closeness, affectionate behavior and satisfaction with dyadic coping were significantly associated with higher odds for couples’ sexual activity at T2 (Table 4). Patients’ perception of affectionate behavior and partners’ perception of affectionate behavior, satisfaction with dyadic coping and emotional closeness were significantly associated with higher odds for sexual activity at T3 (Table 5).

### Sensitivity Analyses of Relationship-Related Variables

Sensitivity analyses on the associations between sexual activity and patients’ and partners’ perceptions of the relationship-related variables with additional adjustment for the respective other partner’s score showed similar results to the main analyses, although with some changes in the significance level (Supplementary Tables A–C). When adjusted for the respective other partner’s score, all baseline associations between patients’ and partners’ relationship experience and sexual activity remained significant (Supplementary Table A). In couples who were sexually active at baseline, patients’ perception of affectionate behavior and partners’ perception of satisfaction with dyadic coping were significantly associated with higher odds for sexual activity at T2 (Supplementary Table B); no significant associations were present in the sensitivity analyses concerning T3 (Supplementary Table C).

## Discussion

### Summary of Main Results

In this large, longitudinal study with 1-year follow-up, roughly 60% of couples were sexually active in the first year after a diagnosis of female breast cancer. At baseline, couples were more likely to be sexually active, if patients and partners felt emotionally close to each other, showed affectionate behavior, or were satisfied with their way of dealing with stress as a couple. These relationship characteristics also predicted sexual activity at follow-up, but only in the group of couples who were sexually active at baseline. Older age and symptoms of depression or low vitality of patients were associated with lower odds for couples being sexually active at baseline. Treatment with chemotherapy was found to influence sexual activity at 5 months follow-up, and only among couples who were sexually inactive at baseline. Thus, individual patient-related and relationship-related characteristics played a role, with the relationship variables being most consistently associated with couples’ sexual activity.

### Relationship-Related Variables

Our results point towards the importance of patients’ and partners’ experience of emotional closeness for couples’ sexual activity. This is in line with findings of Brédart et al. (2011) and with the relationship intimacy model of couple adaptation to cancer, positing that emotional intimacy is central for the experience of relationship well-being in general (Manne and Badr, 2008). In a study by Marino et al. (2017) women’s ratings of emotional closeness to the partner were not related to sexual activity, and most women felt close to their partners. However, the sample consisted of female cancer survivors, who were seen in a specialty clinic for menopause symptoms after cancer. Perhaps personal, partly symptom-related, factors, rather than partner-related factors were central in this specific sub-population, such as being bothered by weight change and not being able to feel like a woman (Marino et al., 2017).

Furthermore, our findings draw attention to additional dimensions of the relationship to consider among couples dealing with breast cancer. Firstly, both patients’ and partners’ reports of affectionate behavior, i.e., kissing and hugging or holding in the couple, were associated with higher odds for sexual activity both at baseline and over time. Of the three relationship-related variables in the present study this rating of behavior is potentially most closely related to the behavioral outcome sexual activity, and couples agree rather strongly on the occurrence of this type of behavior in their relationship (within-couple correlation *r* = 0.68). Previous research has shown that affectionate behavior also is associated with other sexual health indicators, such as satisfaction with sex life (Fisher et al., 2015; Rottmann et al., 2017). However, there is also a relatively large subgroup of couples at baseline that report frequent affectionate behavior but no sexual activity. It is possible that health-related sexual dysfunction may limit their sexual activity, either in relation to breast cancer or in relation to other health- and aging-related issues. Perhaps, for some of these couples, affectionate behavior may be enough. We do not know whether they miss being sexually active or are happy with the situation as it is. This group could be interesting to examine further in future studies.

Secondly, patients’ and partners’ satisfaction with their dyadic coping, i.e., their overall evaluation of how they deal with stress as a couple, was positively associated with sexual activity. This is in line with results of a previous study among university students that also suggested an association between dyadic coping and sexual activity (Bodenmann et al., 2010). High scores on dyadic coping satisfaction may be an indicator of a well-functioning relationship (Falconier et al., 2015). Satisfaction with dyadic coping may also indicate lower stress levels, as the couple is coping well, and this can positively affect sexual activity (Bodenmann et al., 2010).

The baseline findings for the relationship-related variables may underscore the relevance of a positive relationship experience for a couple to engage in sexual activity, which has also been shown in studies based on the general population (Kleinstäuber, 2017). However, the cross-sectional observational design does not allow for conclusions on causality, and the associations are possibly bidirectional. One could, e.g., hypothesize that sexual activity may enhance feelings of emotional closeness in a couple or can be used as a way of dyadic coping with stress.

Importantly, the longitudinal findings differ depending on whether couples are sexually active at baseline or not: The relationship-related variables predict couples’ sexual activity over time, but only in couples who are sexually active at baseline. Thus, feeling emotionally close to one’s partner, showing affectionate behavior and satisfaction with dyadic coping at baseline seem to be protective factors for keeping up sexual activity, but do not contribute to couples taking up sexual activity. Perhaps not the baseline factors *per se*, but changes in these factors are associated with resumption of sexual activity. For example, emotional closeness or satisfaction with dyadic coping may increase in some couples as they go through the cancer trajectory together, which could result in resumption of sexual activity. This could be examined further in future studies.

Furthermore, although the results of our sensitivity analyses do not change the overall conclusions, they indicate that patients’ and partners’ unique perceptions of the relationship constructs may contribute differently to sexual activity. The analyses suggest that the patient’s rating of affectionate behavior and the partner’s rating of satisfaction with dyadic coping may be particularly important for the couple’s sexual activity at T2. These processes within couples could be explored in future research, e.g., using a Dyadic Score Model (Iida et al., 2018), which could examine the contribution that the dyadic level of the relationship-related variables or differences in patients’ and partners’ scores make in predicting the outcome. In-depth knowledge of such processes would be helpful for health professionals such as sexologists or psychologists, who work in-depth with couples.

### Individual Patient and Partner Characteristics

Of the quality of life-related variables patients’ depressive symptoms and lower levels of vitality seemed to affect couples’ sexual activity at baseline, which is in line with previous research (Fobair et al., 2006; Marino et al., 2017; Avis et al., 2018), whereas partners’ depressive symptoms were not associated with couples’ sexual activity. Potentially, the patients’ emotional distress is more important for couples’ sexual activity several months after diagnosis. This could be further examined in future studies. Interestingly, patients’ body image and pain in the arm, breast or shoulder area were not related to couples’ sexual activity. This might partly be explained by the low levels of body image concerns and pain in our sample.

Our study confirms findings suggesting that age is a relevant factor impacting sexual activity after breast cancer diagnosis (Avis et al., 2018). In our study, patients’ older age was associated with lower odds of sexual activity both at baseline and at 5 months-follow up in the subgroup of couples who were not sexually active at baseline.

Among the treatment-related variables, treatment with chemotherapy was negatively associated with sexual activity at 5 months follow-up among couples who were sexually inactive at baseline, which confirms findings from an earlier study among breast cancer survivors recruited within 8 months of cancer diagnosis (Avis et al., 2018). In our study, the side effects of chemotherapy might still have been present at 5 months follow-up, and patients and partners may have been in the process of adjusting to the experience of chemotherapy, although patients usually have completed chemotherapy at this timepoint. The lack of significant associations between treatment-related variables and sexual activity at baseline may be due to heterogeneity in the timing of treatment and questionnaire completion in the sample. The baseline questionnaire was mailed to patients within 4 months after surgery, where patients may have been in different treatment phases, e.g., with some only being about to initiate chemotherapy treatment. In line with previous research, type of surgery and endocrine treatment were not associated with sexual activity (Fobair et al., 2006; Marino et al., 2017; Avis et al., 2018).

Comorbidity of patients and partners was not significantly associated with couples’ sexual activity, although previous research has shown that the presence of medical or chronic conditions is related to less sexual activity (Kleinstäuber, 2017). This difference may potentially be explained by the broad measure we used. Our calculation of the Charlson index is based on registration of diagnoses in relation to hospital visits. It does not include functional impairment or the subjective experience of an illness, which may be more likely to impact sexual activity.

### Sexual Activity in the Study Sample

In the present study, 59, 61 and 62% of couples were sexually active at ≤4 months after diagnosis (T1), and 5 (T2) and 12 months (T3) later, respectively. In approximately 17% of couples, sexual activity status changed from T1 to T2 and from T1 to T3.

Sexual activity was measured using a subjective approach, which did not further define sexual activity. This approach allows respondents to include the aspects that are personally important and meaningful to them. We assessed sexual activity at the couple level, which has the advantage of including both the patient’s and the partner’s perspective. Only couples in whom both the patient and the partner reported sexual activity were categorized as sexually active, which was a rather conservative approach. However, the within-couple agreement was high, and our rates of sexual activity of roughly 60% are on par with other studies of partnered women dealing with breast cancer who reported on sexual activity in the past month: In a study of younger women aged 22–50, who were primarily 2–7 months after diagnosis, 67% reported sexual activity (Fobair et al., 2006). In a study of women with a mean age of 54 years, 52, 59 and 61% reported sexual activity within the first 8 months of diagnosis, and 6 and 18 months later, respectively (Avis et al., 2018).

The rates of sexual activity in our study are lower than what has been found in the Danish population, where only 11% of persons in a relationship reported that they had not had sex with a partner during the past year (Frisch et al., 2019). However, these data from the general population are based on a sample of 15–89-year old persons and, in general, rates of sexual activity decline with older age (Frisch et al., 2019). A study of middle-aged women (mean age 56 years) found, e.g., that 71% had been sexually active in the past year (Addis et al., 2006). In a study of men who were cohabiting with a partner, approximately 75% of the 61–70 year old and almost 50% of men older than 70 reported sexual activity (Beutel et al., 2018).

### Study Strengths and Limitations

The present study has several strengths. To our knowledge it is the first large, longitudinal study examining sexual activity after breast cancer that systematically includes both the patients’ and the partners’ perspective. Eligible couples were identified through nationwide population-based registries. Couples were followed throughout the first year after diagnosis of breast cancer. A broad range of variables was assessed including demographic and health-related, quality of life-related and relationship-related variables, and the study combines self-report data with information from nationwide Danish registries. By using clinical information on breast cancer treatment and other health-related information from nationwide registries, which were established independently of the study, recall and selection bias were avoided with respect to the measurement of these variables. Furthermore, to address issues of multiple testing we only concluded on results that were significant at the 0.01 level.

The study also has several limitations. The relatively low response rates of 32, 24 and 21% at T1, T2 and T3, respectively, might have introduced non-response bias. However, it is challenging to recruit couples into studies (Dagan and Hagedoorn, 2014), and our population-based design permitted us to compare participants with non-participants. We have previously shown that participation in the DCBCC was reduced by lower socioeconomic status, older age and partner morbidity (Terp et al., 2015). This pattern is in line with findings from previous studies on participation in research among cancer patients and their partners (Geller et al., 2011; Christie et al., 2013). In the present study, non-responding couples, i.e., those who did not provide information on sexual activity status, were older and more partners had morbidity. Perhaps non-response is related to the fact that sexuality often is perceived as a sensitive topic, which may be particularly true for elderly people. Possibly, some found these questions to be less relevant at older age, which would be in line with the finding of declining sexual activity at older age (Frisch et al., 2019). However, we do not know if our results can be generalized to populations with more diverse sociodemographic profiles.

We did not assess couples’ sexual activity status prior to diagnosis and can thus not examine the impact of breast cancer diagnosis on their sexual activity, but due to the longitudinal design it was possible to examine change and factors affecting sexual activity in the first year after diagnosis. The subjective assessment of sexual activity does not provide insight into respondents’ concrete understanding of sexual activity. We believe that respondents were primed to think about sexual activity with the partner and not solitary sexual activity, as the question on sexual activity was posed in the last part of a couple-based survey after a range of measures with focus on the couple relationship; however, we cannot be certain about this. Further, some respondents might include affectionate behavior such as kissing or hugging in their understanding of sexual activity. In a recent population-based survey of sex in Denmark, sex was defined as vaginal intercourse, oral sex, anal sex or hand sex (Frisch et al., 2019). Although this does not represent participants’ subjective understanding, we believe most people would spontaneously think of these behaviors when answering questions on their sexual activity in a questionnaire. The assessment of sexual activity can be considered a proxy measure, as it was assessed through one response option on an item assessing satisfaction with sex life.

Furthermore, several of the variables in the questionnaire were measured by single items only. However, the brevity of the measures permitted us to include measurements of a broader range of different constructs.

The use of global, retrospective self-report measures may induce recall bias and may not be optimal to examine how behavior changes and develops in real life settings. Future studies could apply ecological momentary assessments, which allow the study of microprocesses that influence behavior in real-world contexts and maximize ecological validity (Shiffman et al., 2008). However, while the present study does not study microprocesses within couples, it contributes with knowledge on risk or protective factors at a more global level.

Finally, the data were collected approximately 10 years ago and may not mirror recent advances in breast cancer treatment. Nevertheless, a recent review of reviews suggests that breast cancer treatment and its side effects, such as pain and fatigue, as well as psychological issues still affect patients’ quality of life, and that issues related to sexual function need more attention (Mokhatri-Hesari and Montazeri, 2020).

### Clinical Implications

The results of the present study suggest that relationship variables are important for couples’ sexual activity. Thus, a couple perspective should be included in sexual counseling during cancer treatment and rehabilitation. The findings point towards concrete aspects of the couple relationship that clinicians can work with in sexual counselling of patients, partners, and couples. These include working with couples on retaining emotional closeness in the relationship; encouraging couples to use affectionate behavior in their everyday, such as kissing, hugging, and holding each other; and teaching them skills to effectively deal with stress as a couple if needed.

Furthermore, our findings indicate that clinicians should address patients’ emotional distress and fatigue in relation to sexual activity, as well as the role of age and chemotherapy treatment especially during encounters with couples who are not sexually active.

Sexuality or sexual side effects in relation to cancer are not always addressed during oncology treatment (Flynn et al., 2012), and couples may have unmet sexual information and support needs (Gilbert et al., 2016). According to our results, information and counseling about sexuality should already be placed in the treatment phase, as several factors assessed in the first months after diagnosis affected sexual activity throughout the first year after cancer diagnosis. However, we believe it is important to take couples’ individual preferences for timing into account.

One step towards a couple-based approach in sexual counseling is to include the patients’ partner in consultations. In previous research most women diagnosed with breast cancer described a conversation with a professional together with their partner as preferred method of communication about sexuality and intimacy (Den Ouden et al., 2019). Couple-based psychosexual interventions have shown promising results among couples dealing with breast cancer (Carroll et al., 2016), and our findings contribute to consolidate the knowledge base of such interventions.

### Conclusions and Perspectives

In conclusion, this study indicates that not only the patients’ but also the partners’ experience of an affectionate, emotionally close relationship with satisfying dyadic coping is associated with couples’ sexual activity in the first months after breast cancer diagnosis and over time in the re-entry and early survivorship phases. Older age and chemotherapy treatment of patients reduce the odds of couples taking up sexual activity. Patients’ emotional distress and fatigue was associated with lower odds for sexual activity in the first months after diagnosis.

Future research should focus on couples who are currently not sexually active but wish to take up sexual activity. The present study has identified risk factors that may hinder couples in taking up sexual activity over time, but more knowledge on understanding modifiable factors would be important. Furthermore, sexual activity may not be equally important for all couples, and other subjective dimensions, such as satisfaction with sexual life or intimacy among those who are not sexually active, should also be considered.

## Data Availability Statement

The datasets presented in this article are not readily available because we are according to the EU and Danish data protection legislation not allowed to submit the data or give access to the data used for the analyses. Data from the Danish National Patient Register and the Civil Registration System are available from the Danish Health Data Authority^1^ for researchers who meet the criteria for access to confidential data. Data from the clinical database Danish Breast Cancer Cooperative Group^2^ are available for researchers who meet the criteria for access to these confidential data. Requests to access the datasets should be directed to NR, nrottmann@health.sdu.dk

## Ethics Statement

Ethical review and approval was not required for the study on human participants in accordance with the local legislation and institutional requirements. The patients/participants provided their written informed consent to participate in this study.

## Author Contributions

NR, DGH, CJ, and MH contributed to the study design. PVL analyzed the data. NR wrote the first draft of the manuscript. All authors critically revised the manuscript, read and approved the submitted version.

## Funding

The study was funded by the Danish Cancer Society.

## Conflict of Interest

The authors declare that the research was conducted in the absence of any commercial or financial relationships that could be construed as a potential conflict of interest.

## Publisher’s Note

All claims expressed in this article are solely those of the authors and do not necessarily represent those of their affiliated organizations, or those of the publisher, the editors and the reviewers. Any product that may be evaluated in this article, or claim that may be made by its manufacturer, is not guaranteed or endorsed by the publisher.

## Footnotes

^1^ https://sundhedsdatastyrelsen.dk/da/english

^2^ http://www.dbcg.dk/
