# Supplementary material for: Sexual Activity in Couples Dealing With Breast Cancer. A Cohort Study of Associations With Patient, Partner and Relationship-Related Factors
Source: Front Psychol. 2022 Apr 7;13:828422. doi: 10.3389/fpsyg.2022.828422 (PMC9021795; doi:10.3389/fpsyg.2022.828422)
Supplement: Supplementary file 1 [file Data_Sheet_1.pdf]

Supplementary Material

Table A. Associations between patients' and partners' relationship factors and sexual activity at baseline adjusting for the respective other partner's score, n=722

	OR_{crude}	OR_{adj} (95%-CI)[a]	OR_{SDadj} (95%-CI)[a]
**PATIENT**			
**Relationship factors**			
Satisfaction with dyadic coping[b]	1.19***	1.16 (1.06, 1.27)**	1.33 (1.12, 1.57)**
Emotional closeness[b]	1.75***	1.61 (1.29, 2.01)***	1.46 (1.23, 1.75)***
Affectionate behavior (ref. infrequent (<3)): frequent (≥3)	3.00***	1.90 (1.24, 2.91)**	1.90 (1.24, 2.91)**
**PARTNER**			
**Relationship factors**			
Satisfaction with dyadic coping[b]	1.13**	1.12 (1.03, 1.23)**	1.24 (1.06, 1.48)**
Emotional closeness[b]	1.69***	1.57 (1.24, 1.98)***	1.43 (1.19, 1.72)***
Affectionate behavior (ref. infrequent (<3)): frequent (≥3)	3.49***	2.69 (1.76, 4.11)***	2.69 (1.76, 4.11)***

Abbreviations: OR, Odds Ratio; OR_{SD}, Odds Ratio measured in units of sample SD for continuous covariates

[a]Adjusted for patient age, type of surgery, chemotherapy and the respective other partner's covariate

[b]Per scale unit (resp. SD) increase

*p<0.05, **p<0.01, ***p<0.001

Table B. Associations between patients' and partners' relationship factors and sexual activity at time T2, adjusting for the respective other partner's score and stratified on sexual activity at baseline, n=533

	Couples not sexually active at time T1, n=210			Couples sexually active at time T1, n=323		
	OR_{crude}	OR_{adj} (95%-CI)[a]	OR_{SDadj} (95%-CI)[a]	OR_{crude}	OR_{adj} (95%-CI) [a]	OR_{SDadj} (95%-CI)[a]
**PATIENT**						
**Relationship-related factors**						
Satisfaction with dyadic coping[b]	1.14	1.14 (0.96, 1.37)	1.33 (0.91, 2.00)	1.28*	1.16 (0.93, 1.46)	1.29 (0.88, 1.90)
Emotional closeness[b]	1.07	0.94 (0.61, 1.46)	0.95 (0.64, 1.41)	2.01**	1.75 (1.08, 2.86)*	1.48 (1.06, 2.09)*
Affectionate behavior (ref. infrequent (<3)): frequent (≥3)	1.62	1.37 (0.62, 3.33)	1.37 (0.62, 3.33)	6.48***	5.52 (2.15, 14.14)***	5.52 (2.15, 14.14)***
**PARTNER**						
**Relationship-related factors**						
Satisfaction with dyadic coping[b]	1.11	1.10 (0.92, 1.32)	1.22 (0.84, 1.79)	1.39***	1.36 (1.12, 1.65)**	1.69 (1.21, 2.34)**
Emotional closeness[b]	1.45	1.64 (1.01, 2.67)*	1.64 (1.01, 2.67)*	1.93**	1.54 (0.91, 2.59)	1.30 (0.95, 1.77)
Affectionate behavior (ref. infrequent (<3)): frequent (≥3)	2.05*	2.09 (0.97, 4.52)	2.09 (0.97, 4.52)	3.69**	1.53 (0.56, 4.16)	1.53 (0.56, 4.16)

Abbreviations: OR, Odds Ratio; OR_{SD}, Odds Ratio measured in units of sample SD for continuous covariates

[a]Adjusted for patient age, type of surgery, chemotherapy and the respective other partner's covariate

[b]Per scale unit (resp. SD) increase

*p<0.05, **p<0.01, ***p<0.001

Table C. Associations between patients' and partners' relationship factors and sexual activity at time T3, adjusting for the respective other partner's score and stratified on sexual activity at time T1, n=471

	Couples not sexually active at time T1, n=181			Couples sexually active at time T1, n=290		
	OR_{crude}	OR_{adj} (95%-CI)[a]	OR_{SDadj} (95%-CI)[a]	OR_{crude}	OR_{adj} (95%-CI)[a]	OR_{SDadj} (95%-CI)[a]
**PATIENT**						
**Relationship-related factors**						
Satisfaction with dyadic coping[b]	1.03	0.97 (0.82, 1.14)	0.94 (0.65, 1.33)	1.32*	1.22 (0.92, 1.62)	1.40 (0.87, 2.27)
Emotional closeness[b]	1.12	1.02 (0.68, 1.52)	1.02 (0.71, 1.46)	1.53	1.22 (0.70, 2.14)	1.15 (0.78, 1.70)
Affectionate behavior (ref. infrequent (<3)): frequent (≥3)	1.47	1.06 (0.49, 2.31)	1.06 (0.49, 2.31)	4.69**	3.15 (1.03, 9.65)*	3.15 (1.03, 9.65)*
**PARTNER**						
**Relationship-related factors**						
Satisfaction with dyadic coping[b]	1.14	1.17 (0.98, 1.40)	1.39 (0.96, 2.02)	1.37**	1.32 (1.06, 1.65)*	1.60 (1.10, 2.34)*
Emotional closeness[b]	1.27	1.29 (0.83, 1.99)	1.29 (0.83, 1.99)	2.08**	1.94 (1.10, 3.43)*	1.49 (1.06, 2.09)*
Affectionate behavior (ref. infrequent (<3)): frequent (≥3)	1.98*	1.97 (0.93, 4.17)	1.97 (0.93, 4.17)	4.37**	2.23 (0.12, 331.67)	2.23 (0.12, 331.67)

Abbreviations: OR, Odds Ratio; OR_{SD}, Odds Ratio measured in units of sample SD for continuous covariates
[a]Adjusted for patient age, type of surgery, chemotherapy and the respective other partner's covariate
[b]Per scale unit (resp. SD) increase
*p<0.05, **p<0.01
